# Inflammation as a Link between Obesity and Metabolic Syndrome

**DOI:** 10.1155/2012/476380

**Published:** 2012-03-01

**Authors:** Faloia Emanuela, Michetti Grazia, De Robertis Marco, Luconi Maria Paola, Furlani Giorgio, Boscaro Marco

**Affiliations:** Division of Endocrinology, Polytechnic University of Marche, via Conca 71, 60126 Ancona, Italy

## Abstract

The metabolic syndrome is a complex of clinical features leading to an increased risk for cardiovascular disease and type 2 diabetes mellitus in both sexes. Visceral obesity and insulin resistance are considered the main features determining the negative cardiovascular profile in metabolic syndrome. The aim of this paper is to highlight the central role of obesity in the development of a chronic low-grade inflammatory state that leads to insulin resistance, endothelial and microvascular dysfunctions. It is thought that the starting signal of this inflammation is overfeeding and the pathway origins in all the metabolic cells; the subsequent increase in cytokine production recruits immune cells in the extracellular environment inducing an overall systemic inflammation. This paper focuses on the molecular and cellular inflammatory mechanisms studied until now.

## 1. Introduction

Metabolic syndrome represents one of the major public health challenges worldwide. Different definitions are available describing overlapping but not identical population [1]. The first description goes back to 1988 when Reaven described Syndrome X as the association of insulin resistance, elevated glucose, hypertension, low HDL cholesterol, and augmented VLDL triglycerides [2]. However he did not include obesity, now identified as one of the essential criterion, especially visceral obesity [1].

Overweight and obesity progress to metabolic syndrome through pathophysiological mechanisms at the moment largely unclear. It has been hypothesized that the state of chronic low-grade inflammation associated with excess adipose tissue may explain the development of the obesity-related pathologies, such as type 2 diabetes mellitus and cardiovascular disease. This inflammatory response is different from the classical responce defined by the cardinal signs of redness, swelling, heat, and pain [3, 4]. Furthermore, it plays an important role in the development of insulin resistance that triggers the associated comorbidities of metabolic syndrome, such as atherosclerosis, dyslipidemia, hypertension, prothrombotic state, and hyperglycemia [5–8].

## 2. Metabolic Syndrome

### 2.1. Prevalence and Definition

The metabolic syndrome is identified as a condition of increased risk for cardiovascular disease (CVD) and type 2 diabetes mellitus (T2DM) in both sexes. Subjects with metabolic syndrome have three times risk of suffering a heart attack or stroke, twice of dying from such an event, and fivefold greater risk of developing type 2 diabetes mellitus when compared to people without the metabolic syndrome [9]. 

It was first described in 1920 when Kylin, a Swedish physician, demonastrated the association of high blood pressure (hypertension), high blood glucose (hyperglycaemia), and gout [10]. Later in 1947, Vague described that the visceral obesity was commonly associated with the metabolic abnormalities found in CVD and T2DM [11].

The prevalence of metabolic syndrome varies depending on the definition applied, the ethnicity, and the age of the study population. The two currently used definitions are that of the American Heart Association/National Heart, Lung, and Blood Institute (AHA/NHLBI) and the other one of the International Diabetes Federation (IDF). They describe overlapping but not identical populations. The major difference is that the first one sets the presence of three of five possible components, whereas the second one identifies in the waist circumference, and therefore in the abdominal obesity, the mandatory diagnostic criterion (Table 1) [12, 13].

Both AHA/NHLBI and IDF recognize the need of a variable definition of elevated waist circumference among different populations. The IDF suggests for Europids a threshold for increased waist circumference of at least 94 cm in men and 80 cm in women; whereas the AHA/NHLBI defines for the US population the cutoff of at least 102 cm for men and 88 cm for women (Table 1) [12, 13]. Two important studies show the rationale for using different cut-off points of waist circumference in people of Asian extraction [14, 15]. East Asian and South Asian populations may have significant differences in lipid indices, fat mass as a proportion of BMI and cardiovascular morbidity. More studies are necessary to clarify these differences before consensus on separate cutoffs for waist circumference will be established for these ethnic groups [16].

## 3. Metabolic Syndrome as a Risk Condition

It is evident that a condition characterized by multiple risk factors will carry a greater risk for adverse clinical outcomes.

The so-called classic risk factors of cardiovascular disease (CVD) and coronary heart disease (CHD) include many of the components of the metabolic syndrome. The most widely applied prediction equation is the Framingham risk score, less well validated for persons with T2DM rather than without T2DM [17]. More recently, Oxford investigators have developed a risk engine based on the large UK Prospective Diabetes Study (UKPDS) database with validated CVD risk estimate for people with T2DM [18–20]. Both methods take into consideration clinical parameters, as well as age, smoking, blood pressure, and serum lipid levels. The UKPDS risk engine also includes duration of diabetes and plasma glucose levels.

## 4. Obesity

### 4.1. Prevalence and Definition

Obesity is a metabolic disease of pandemic proportion. The World Health Organization estimates that 300 million of adults worldwide are obese and more than 1 billion are overweight [21].

Obesity is commonly classified into subgroups depending on suspected etiology: monogenic obesity, syndromic obesity, and polygenic or common obesity [22].

The monogenic obesity is an autosomal form characterized by an extremely severe obesity in the absence of developmental delays; there are about 20 single gene disruptions that result in an autosomal form of obesity [23]. Interestingly, all these mutations position the leptin/melanocortin pathway in the central nervous system (CNS) as critical in the regulation of whole-body energy homeostasis, and obesity in these cases appears to be the result of increased appetite and diminished satiety [24].

Syndromic obesity arises from discrete genetic defects or chromosomal abnormalities at several genes, and it can be autosomal or X-linked. They are clinically obese subjects additionally distinguished by mental retardation, dysmorphic features and organ-specific developmental abnormalities; one of the most well-known forms of syndromic obesity is Prader-Willi syndrome [22].

The most common form of obesity, which affects the general population, is the polygenic form resulting from a long-term positive energy balance; the energy excess is stored in adipose tissue and, if this process is prolonged, obesity develops. The balance between energy intake and expenditure is influenced by a complex interplay of genetic, environmental, and social factors. In common obesity, some yet unclear signals lead to insulin resistance and to health risks, such as increased risk of CVD [25].

A positive energy balance or obesity can also be secondary to systemic disorders: hypothyroidism diminishes energy need, insulinoma causes obesity by promoting energy intake via recurrent hypoglycemia, and Cushing disease is associated with obesity of the classical centripetal type. Other etiological factors of obesity include the binge eating disorder, a high glycemic diet, a sedentary lifestyle, and use of certain medications like psychotropic drugs [26].

Obesity is a potent risk factor for metabolic and cardiovascular disease at the population level. At the individual patient level, however, correlations between body mass index and cardiovascular disease are not always straightforward due, in part, to differences among adipose tissue depots with respect to the overall rate of adipocyte dysfunction, tissue vascularization, and local degree of inflammation. Adipose tissue develops in several distinct anatomical depots within the body, and the differential expansion of these depots is of great importance. Expansion of visceral or abdominal white adipose tissue (WAT) has been most strongly correlated to insulin resistance and cardiovascular disease in humans and animals. Several studies have documented that peripheral adiposity (especially leg fat) may protect against cardiovascular risk [27, 28].

## 5. Obesity and Inflammation

One challenge aspect of metabolic syndrome is understanding the cellular mechanisms that link the metabolic abnormalities with the pathophysiological effects that later generate clinical disease.

The link between obesity and inflammation has been derived from the finding that proinflammatory cytokines are overexpressed in obesity [29]. 

Adipose tissue is an heterogeneous mix of adipocytes, stromal preadipocytes, immune cells, and endothelium, and it can respond rapidly and dynamically to alterations in nutrient excess through adipocyte hypertrophy and hyperplasia [30]. With obesity and progressive adipocyte enlargement, the blood supply to adipocytes may be reduced with consequent hypoxia [31]. Hypoxia has been proposed to be an inciting etiology of necrosis and macrophage infiltration into adipose tissue that leads to a overproduction of proinflammatory factors like inflammatory chemokines. This results in a localized inflammation in adipose tissue that propagates an overall systemic inflammation associated with the development of obesity-related comorbidities [32]. This paper will focus on three adipokine produced by macrophages: tumor necrosis factor-alpha (TNF-*α*), interleukin-6 (IL-6), and adiponectin [33].

*TNF-*α**. It is a proinflammatory cytokine that exerts numerous effects in adipose tissue including lipid metabolism and insulin signaling whose circulating levels are increased with obesity and decreased with weight loss. An increase in TNF-*α* promotes the secretion of other proinflammatory cytokines IL-6 and TNF-*α*, and reduces anti-inflammatory cytokines like adiponectin [34]. Evidence suggests that TNF-*α* induces adipocytes apoptosis [35] and promotes insulin resistance by the inhibition of the insulin receptor substrate 1 signaling pathway [36].

*IL-6*.The primary source of circulating IL-6 is macrophages that have infiltrated WAT; IL-6 has an important role in the regulation of whole-body energy homeostasis and inflammation. Both in vitro and in vivo studies have confirmed that IL-6 is capable of suppressing lipoprotein lipase activity. IL-6 receptor is also expressed in several regions of the brain, such as the hypothalamus, in which it controls appetite and energy intake [37].

*Adiponectin*. Weight loss has been shown to increase adiponectin levels; in animal models of obesity and insulin resistance, its levels are reduced. Adiponectin regulates lipid and glucose metabolism, increases insulin sensibility, regulates food intake and body weight, and protects against chronic inflammation [38]. Human studies show that hypoadiponectinemia is associated with insulin resistance, hyperinsulinemia, and the possibility of developing type 2 diabetes, independent of fat mass [39].

Furthermore, more recent studies have been focused on the intracellular pathways of inflammation. In obesity, it is thought that the starting signal of inflammation is overfeeding and the pathway origins in all the metabolic cells, for example, in the adipocyte, hepatocyte, or myocyte. Studies in mice and humans evidence that consumption of nutrients may acutely evoke inflammatory responses [40, 41]. Metabolic cells, such as adipocytes, respond to this insult beginning the inflammatory response. In obese men and women, if compared with lean controls, adipose tissue and liver display an increased activation of three kinases able to induce the expression of inflammatory cytokines: the c-jun N-terminal kinase (JNK), the inhibitor of k kinase (IKK), and the protein kinase R (PKR) [42, 43]. In the same metabolic tissues, the inflammasome and the Toll-like receptors (TLRs) of the innate immune system are also activated [44–46]. Nutrients or inflammatory signals may activate the TLRs pathways and downstream JNK, IKK, and PKR. These kinases regulate downstream transcriptional programs through the transcription factors activator protein-1 (AP-1), NF-*κ*B, and interferon regulatory factor (IRF), inducing upregulation of inflammatory mediator gene expression. The increase in cytokines exacerbates receptor activation by establishing a positive feedback loop of inflammation and the inhibitory signaling of metabolic pathways [8].

The hypothesis is that nutrients are *not self* and therefore elicit an immune response when metabolized, or they are naturally associated with inflammatory molecules released into the circulation [47, 48]. In lean healthy animals, a low pulsatile inflammatory response occurs during the feeding and resolves after the nutrients are metabolized [40, 41]. In obesity or in overfeeding, responses become more intense and resolution less efficient. These signals accumulate over time and may reach a level where the professional immune cells are recruited and activated leading to an unresolved inflammatory response within the tissue [43, 45]. The quality of diet may produce different responses: a diet rich in fruit and fibre is reported to not induce significant inflammation compared to an equicaloric high-fat diet [49].

## 6. Insulin Resistance and Endothelial/Microvascular Dysfunction

Inflammation in obesity results in the inhibition of the insulin receptor signaling cascade: the three kinases described above, JNK-IKK-PKR, can target insulin receptor substrate 1 (IRS-1) for serine phosphorilation and degradation [6–8].

Insulin has important effects on the endothelium, increasing nitric oxide (NO) availability and stimulating vasodilatation [50]. In contrast, insulin resistance is associated with endothelial dysfunction. [51, 52].

Endothelial and microvascular dysfunction are present in obese subjects and represent important factors in metabolic disturbances, since they could influence both vascular resistance and insulin-mediated glucose disposal, contributing to hypertension and insulin resistance in obesity [52, 53].

Endothelial dysfunction is an early process in obesity: it is present even in the absence of hypertension or hyperglycemia, and it is associated with visceral obesity suggesting that obesity is an independent risk factor. It is characterized by impaired endothelium-dependent vasodilatation, reduced arterial compliance, and accelerated process of atherosclerosis [54].

It has been hypothesised an inflammatory aetiology for both obesity and atherosclerosis [55–57]. Immune cells play an important role in all stages of the atherosclerotic process [58]; in addiction, a reduction in NO, a key regulator of endothelial homeostasis, and an increase in reactive oxygen species result in endothelial dysfunction and a proatherogenic vascular bed [59].

Therefore, Gavin and collegues demonstrated a microvascular dysfunction in obese subjects resulting in a reduction on capillary density in skeletal muscle and skin when compared to lean individuals. This produces a blunted response to vasodilatation induced by oral glucose loading probably due to impaired capillary recruitment in response to an increased plasma insulin level. There is also a reduction in transcapillary delivery of insulin to muscle in obese subjects [60].

## 7. The Effect of Different Therapeutic Approaches on Inflammatory Markers

Considering the obesity-induced inflammatory state, studies from the literature have evaluated therapeutic interventions by interfering with inflammatory mediators.

In patients with type 2 diabetes mellitus, the pancreatic IL1-receptor antagonist (IL-1Ra) expression is reduced and high glucose concentrations induce IL-1 production in *β*-cells leading to impaired insulin secretion, decreased cell proliferation, and apoptosis. Larsen et al., using anakinra, a recombinant human IL-1Ra, in 70 patients with type 2 diabetes mellitus, observed after 13 weeks an improved *β*-cell secretory function (reduced glycated haemoglobin level, enhanced C-peptide secretion, reduced ratio of proinsulin to insulin) and a reduction of IL-6 and C-reactive protein, markers of systemic inflammation [61]. The same authors in a 39-week follow-up study investigated the durability of these responses: the reduced proinsulin/insulin ratio and CRP and IL-6 serum levels were maintained. The improvement in *β*-cell function could be a consequence of inhibited IL-1 signaling and not only of improved glycaemia per se [62].

In obese humans are observed increased circulating levels of TNF-*α*; this event has been proposed to be causatively involved in the evolution of insulin resistance, type 2 diabetes, and its complications.

Animal studies showing that interference with TNF-*α* signaling protects against developing the metabolic syndrome in obesity and studies in patients with chronic inflammatory conditions, such as rheumatoid arthritis and psoriasis, clearly show that quenching TNF-*α* activity improves insulin sensitivity [63, 64].

Alternatively, some studies were conducted to demonstrate the effect of TNF-*α* neutralization on insulin sensitivity in patients with type 2 diabetes: most of them indicated no appreciable effect of TNF-*α* neutralization on insulin sensitivity [65–67].The basis for this controversy is unclear but may relate to patient populations studied or length of clinical trials; all these studies potentially did not allow sufficient time for normalization of the metabolic derangements. In fact more recently, a long-term study conducted in obese subjects with glucose alterations and subclinical inflammation treated with etanercept, TNF-*α* antagonist, found an improved fasting glucose, increased ratio of high molecular weight (HMW) adiponectin to total adiponectin, and decreased soluble intracellular adhesion molecule-1 (sICAM) [68].

However, this evidence brings to question whether in TNF-*α* is a causative link between adiposity and insulin resistance [69].

The thiazolidinediones (TZDs), a class of potent agonists of peroxisome proliferator activated receptor-*γ* (PPAR*γ*), increasing the activation of this transcription factor in adipose tissue, restores lipogenic function and decrease inflammation [70]. TZDs also block the ability of TNF-*α* to alter the most proximal steps of insulin signaling through the serine phosphorylation of insulin receptor and increase adiponectin expression [71]. One in vitro study demonstrated that adiponectin exerts potent immunosuppressive properties inducing the production of anti-inflammatory mediators IL-10 and IL-1 receptor antagonist (IL-1Ra) in a variety of myeloid cell types. IL-10 can inhibit the production of many other proinflammatory cytokines including IL-1, IL-2, INF*γ*, and TNF-*α* and impairs the phagocytic and all-stimulatory capacity of macrophages [72].

In addition, adiponectin through the upregulation of IL-10 increases the tissue inhibitor metalloproteinase-1 (TIMP-1) levels in human macrophages preventing the extracellular degradation [73].

## 8. Conclusions

**Figure 1:**
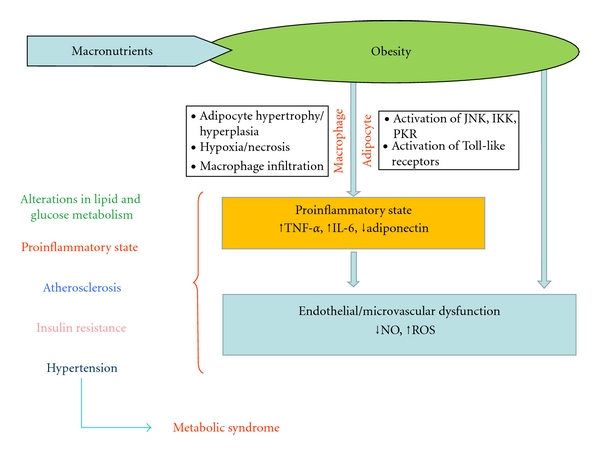
Mechanisms linking abdominal obesity and metabolic syndrome. TNF-*α* : tumour necrosis factor alpha; IL-6 : interleukin 6; NO : nitric oxide; ROS : reactive oxygen species; JNK : c-jun N-terminal kinase; IKK : Inhibitor of k kinase; PKR : protein kinase R.

The association between visceral obesity and metabolic syndrome is well known, but the pathophysiological mechanisms that explain this link are not completely understood. Metabolic syndrome is a complex of clinical features, the most important of which is an increased visceral fat depot. Obesity results in a proinflammatory state starting in the metabolic cells (adipocyte, hepatocyte, or myocyte) and also recruiting immune cells with the consequent release of inflammatory cytokines (TNF-*α*, IL-6, adiponectin, etc.). It has been hypothesized that the obesity-induced inflammatory process may lead to complications such as hypertension, atherosclerosis, dyslipidaemia, insulin resistance, and diabetes mellitus which characterize metabolic syndrome (Figure 1), but other studies are necessary to focus on the role of adipose tissue in the pathogenesis of diabetes mellitus and cardiovascular disease.

## Figures and Tables

**Table 1:** The most widely used definitions for metabolic syndrome.

AHA/NHLBI [12]: At least three of the following five features	IDF [13]: Elevated waist circumference plus any two of the other features
*Waist circumference *	*Waist circumference *
(i) Non-Asian origin: ≥102 cm in men or ≥88 cm in women	(i) Europids, Sub-Saharan Africans, Middle Eastern: ≥94 cm in men or ≥80 cm in women
(ii) Asian origin (both East and South Asians): ≥90 cm in men or ≥80 cm in women	(ii) both East Asians and South Asians; South and Central Americans: ≥90 cm in men or ≥80 cm in women
	(iii) Japanese: ≥85 cm in men or ≥90 cm in women

*Triglycerides (fasting) * ≥150 mg/dL or on drug therapy for high triglycerides	*Triglycerides (fasting) * ≥150 mg/dL or on drug therapy for high triglycerides

*HDL cholesterol * <40 mg/dL in men or <50 mg/dL in women or on drug therapy for low HDL-C	*HDL cholesterol * <40 mg/dL in men or <50 mg/dL in women or on drug therapy for low HDL-C

*Blood pressure * ≥130 mmHg systolic or ≥85 mmHg diastolic or on drug therapy for hypertension	*Blood pressure * ≥130 mmHg systolic or ≥85 mmHg diastolic or on drug therapy for hypertension

*Glucose (fasting) * ≥100 mg/dL or or on drug therapy for elevated glucose	*Glucose (fasting) * ≥100 mg/dL or or on drug therapy for elevated glucose

AHA/NHLBI: American Heart Association/National Heart, Lung, and Blood Institute; IDF: International Diabetes Federation; HDL: high-density lipoprotein.
